# Sirtuins transduce STACs signals through steroid hormone receptors

**DOI:** 10.1038/s41598-020-62162-0

**Published:** 2020-03-24

**Authors:** Henry K. Bayele

**Affiliations:** Department of Structural and Molecular Biology, Division of Biosciences, University College London, Darwin Building, Gower Street, London, WC1E 6BT United Kingdom

**Keywords:** Hormones, Transcriptional regulatory elements

## Abstract

SIRT1 protects against several complex metabolic and ageing-related diseases (MARDs), and is therefore considered a polypill target to improve healthy ageing. Although dietary sirtuin-activating compounds (dSTACs) including resveratrol are promising drug candidates, their clinical application has been frustrated by an imprecise understanding of how their signals are transduced into increased healthspan. Recent work indicates that SIRT1 and orthologous sirtuins coactivate the oestrogen receptor/ER and the worm steroid receptor DAF-12. Here they are further shown to ligand-independently transduce dSTACs signals through these receptors. While some dSTACs elicit ER subtype-selectivity in the presence of hormone, most synergize with 17β-oestradiol and dafachronic acid respectively to increase ER and DAF-12 coactivation by the sirtuins. These data suggest that dSTACs functionally mimic gonadal steroid hormones, enabling sirtuins to transduce the cognate signals through a conserved endocrine pathway. Interestingly, resveratrol non-monotonically modulates sirtuin signalling, suggesting that it may induce hormesis, i.e. “less is more”. Together, the findings suggest that dSTACs may be informational molecules that use exploitative mimicry to modulate sirtuin signalling through steroid receptors. Hence dSTACs’ intrinsic oestrogenicity may underlie their proven ability to impart the health benefits of oestradiol, and also provides a mechanistic insight into how they extend healthspan or protect against MARDs.

## Introduction

Among the seven human sirtuins, SIRT1 (silent information regulator 2 homologue 1) has received the most attention because of its many roles including gene regulation, genomic stability and energy metabolism^1,2^. SIRT1 is also of enormous interest as a viable drug target because it protects against several conditions including obesity, type 2 diabetes, cancer and cardiovascular and neurodegenerative diseases^3,4^. However, deciphering how this protein is regulated is complicated by the fact that it appears to be a hub for multiple networks while also participating in several reciprocal interactions and autoregulatory loops^5^. This further frustrates our understanding of how it transduces signals from interacting partners but especially from sirtuin activating compounds (STACs) such as resveratrol. These molecules are thought to allosterically activate SIRT1 by directly binding to its N-terminal STACs-activation domain, STACs-AD. Although this proposed mechanism has been intensely controversial^6–8^, evidence suggests that a single residue (E230) within the STACs-AD may be responsible for allosteric activation^9^. Furthermore, co-crystal structures of STACs and SIRT1 support direct binding consistent with an allosteric mechanism^10,11^. Alternative mechanisms propose that AMP-activated protein kinase may transduce STACs signals but the pathway leading to SIRT1 activation is unclear^12,13^. Hence there is no consensus on how SIRT1 and orthologous sirtuins translate the beneficial effects of resveratrol and related dietary STACs (dSTACs) to extend healthspan in diverse organisms including humans.

Since dSTACs can allosterically increase sirtuin deacetylase activities, they portend pharmacological interventions for MARDs^8,14–16^. However this has been slowed by an imprecise understanding of how the cognate allosteric signals are transduced by the sirtuins, partly because dSTACs are functionally promiscuous^8,17^. A clue to how they work *in vivo* may be in the fact that with the exception of SRT1720 and its relatives, all are phytoestrogens^8,17^ that are structurally similar to the oestrogen receptor (ER) steroidal ligand 17β-oestradiol (hereafter referred to as E2 or oestrogen), and have been shown to compete with this hormone for ER binding^18–21^. In what appears to hint at how dSTACs may modulate sirtuin signalling, resveratrol in particular has been shown to activate the ER and to competitively inhibit oestradiol binding to the receptor’s ligand binding domain (LBD)^22^. Recent work^23^ described SIRT1 and the orthologous sirtuins Sir2 (*Saccharomyces cerevisiae*) and Sir-2.1 (*Caenorhabditis elegans*) as nuclear receptor coregulators that coactivate the ER and DAF-12, the steroid receptor that regulates nematode lifespan and reproductive development^24–26^. The data reported herein build on those findings to show that dSTACs ligand-independently enhance sirtuin signalling through these receptors. The results also suggest that these molecules may modulate SIRT1 signalling through the ERs by mimicking oestradiol, thus providing a plausible alternative mechanism by which they may regulate healthspan.

## Results

### Dietary STACs ligand-independently modulate ER coactivation by sirtuins

**Figure 1:**
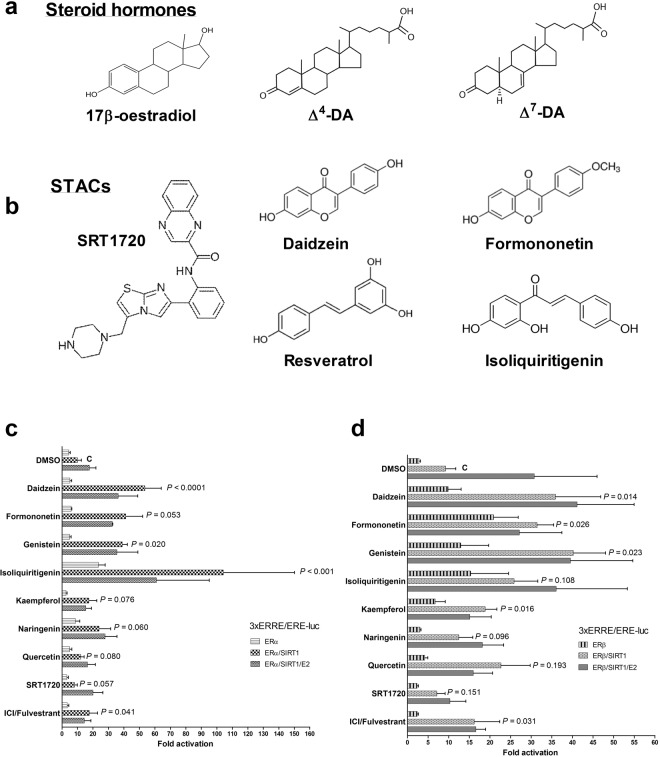
Structural and functional mimicry of steroid hormones by dietary STACs. (**a)** Structural comparison of the steroid hormones oestradiol (of vertebrates) and nematode Δ^4^- and Δ^7^- dafachronic acids (DA) with (**b)**, exemplar dSTACs and the synthetic compound SRT1720. (**c**,**d**) STACs differentially modulate SIRT1 signalling through ERα and ERβ. ERα (**c)** or (**d**) ERβ was cotransfected into Hep3B cells with the ER reporter gene 3x ERRE.ERE-luc, with and without SIRT1. Cells were treated for ~24 h with DMSO (solvent control) or 100 µM of the indicated dSTACs and SRT1720 (1 µM) alone or in combination with 100 nM E2. The pure ER antagonist ICI 182,780/fulvestrant (100 nM) was used as negative control. Reporter activity was normalized to beta galactosidase (βgal) internal control; fold activation was with respect to gene expression in DMSO-treated cells. Graphs show data as means of 3 independent experiments ± S.E.M. Statistical significance was determined by Dunnett’s multiple comparison testing, comparing levels of gene expression induced by DMSO control (C) with each of the dSTACs shown. Actual or indicative *P* values are shown for each compound. Since emphasis was on finding differences between dSTACs compared with DMSO, statistical significance was not tested on differences between DMSO-treated cells and cells simultaneously treated with dSTACs and E2.

Structural comparisons of dSTACs with steroid hormones (Fig. 1a,b) hinted at functional mimicry that might enable sirtuins to transduce dSTACs signals through steroid receptors. Hence based on a recent report^23^, dSTACs were tested to determine if they could modulate sirtuin signalling by acting as oestradiol mimics. ERα or ERβ was expressed in Hep3B cells together with the ER reporter gene, without or with SIRT1. After treating the cells with various subclasses of dSTACs **(**Table 1), they were found to differentially modulate both ERα and ERβ ligand-independently, with the latter being relatively more activated by dSTACs alone than the former; however the activities of both receptors were further enhanced by SIRT1 **(**Fig. 1c,d**)**. Compared with DMSO, the isoflavones and the chalcone isoliquiritigenin induced the strongest ligand-independent ERα coactivation while the flavonols quercetin and kaempferol were the weakest dSTACs. Interestingly, most of these molecules ligand-independently increased SIRT1 coactivation of both receptors but in the presence of oestradiol some (primarily the isoflavones) elicited ER subtype-selectivity, reducing ERα signalling while ERβ transcriptional activation was largely unaffected. Of note, although the pure antioestrogen ICI 182,780 (also known as fulvestrant, a drug used to treat hormone-dependent breast cancer) reduced ERα signalling, ERβ remained partially active consistent with the fact that this drug is a selective ER down-regulator that induces ERα degradation but stabilizes and maintains ERβ in a transcriptionally-active conformation^27–30^. These data are consistent with other findings that that class of phytoestrogens bind and activate ERβ preferentially^18–21^. They also strongly suggest that dSTACs contain structural information (see Supplementary Table S1) that enables them to functionally mimic E2 and to potently modulate SIRT1 signalling through the ERs. However in contrast to dSTACs, the synthetic STAC SRT1720 did not increase ER coactivation by SIRT1 (Fig. 1c,d). While it is possible that its effects may be cell type-specific, these results indicate that SRT1720 signals may be transduced differently i.e. by allostery (direct activation of SIRT1 enzymatic activity) rather than via the ERs. The clue to this may be in the fact that unlike dSTACs which share aromatic nuclei with oestradiol, SRT1720 has an imidazothiazole core.

**Table 1:** Subclasses of natural STACs used in this study and examples of their dietary sources.

Sirtuin Activating Compound (STAC)	Polyphenol subclass	Exemplar dietary sources
Daidzein	Isoflavone	Legumes e.g. soybean; tofu, soymilk
Formononetin	Isoflavone	Soybean, tofu, soymilk
Genistein	Isoflavone	Soybean, tofu, soymilk
Isoliquiritigenin	Chalcone	Licorice
Kaempferol	Flavonol	Tomatoes, kale, leek, green tea, Brussels sprouts, potatoes
Naringenin	Flavanone	Grapefruit, lemon, cherries, cocoa
Quercetin	Flavonol	Onions, cruciferous vegetables (leek, broccoli, cabbage), pepper
Resveratrol	Stilbene	Grapes, red wine, berries, nuts

**Figure 2:**
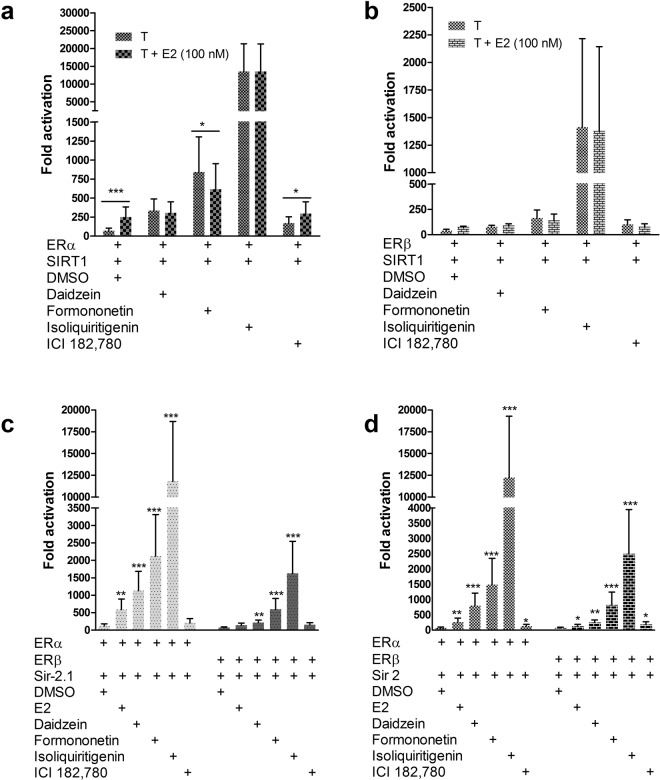
Comparative dSTACs signal transduction by sirtuins through the oestrogen receptors. (**a**) STACs exploit their mimicry of sex hormones to modulate SIRT1 signalling through ERα and (**b)** ERβ. Hep3B cells were cotransfected with ERα or ERβ, 3x ERRE.ERE-luc and with SIRT1. Cells were then treated (T) with DMSO or 100 µM of select dSTACs alone and in combination with 100 nM E2. (**c**) Sir-2.1 and (**d)** Sir2 transduce STACs signals through the ERs. Assay was similar to (**a**,**b**) except that cells were treated with DMSO, 100 nM E2 and 100 µM of select dSTACs or 100 nM ICI 182,780. Data were normalized to βgal internal control with DMSO as the baseline to determine fold activation. Results show means ± S.E.M of 3 independent experiments; paired Student’s *t*-test for differences are significant *(*P* ≤ 0.05), very significant **(*P* ≤ 0.01) and extremely significant ***(*P* ≤ 0.001). Where not shown, these differences are not significant.

Further comparisons showed that independently of oestradiol, isoliquiritigenin most strongly increased ER coactivation by SIRT1 (Fig. 2a,b), Sir-2.1 (Fig. 2c) and Sir2 (Fig. 2d**)**, supporting other findings that it may be a selective ER modulator^31^. Consistent with previous data^23^, in all cases dSTACs stimulated ERα coactivation by the sirtuins more strongly than ERβ, possibly reflecting their differential expression in liver cells^19^. Although it could be argued that these sirtuins may have activated the receptors by deacetylation, that seems unlikely because ERα in particular has been shown to be repressed by deacetylation whereas the data showed that both receptors were transcriptionally competent, suggesting that they were probably acetylated. Furthermore, ER deacetylation and coactivation are distinct both mechanistically and in their outputs^1,8,23,32^; this includes the fact that as coactivators, these sirtuins recruit the ERs through a nuclear receptor box (NR-box) to regulate gene expression^23^. Together these results suggest that these sirtuins may transduce dSTACs signals through steroid receptors independently of allostery or deacetylation. The data also show that dSTACs elicit ER subtype-selectivity in the presence of oestradiol but are also capable of enhancing ER coactivation by SIRT1 even in the absence of hormone (see **Discussion**).

### Dietary STACs differentially induce SIRT1 binding to oestrogen receptors

**Figure 3:**
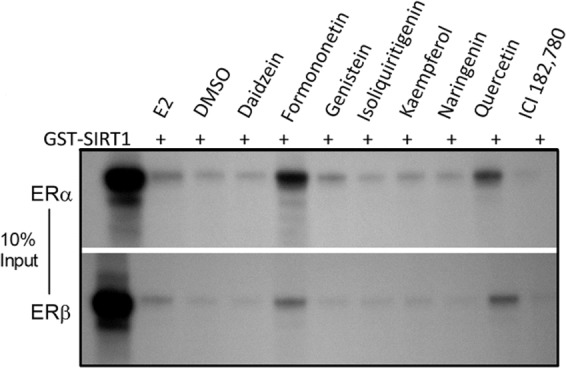
Dietary STACs bind differentially to ER-SIRT1 transcriptional complexes. Pull-down assays were performed with GST-SIRT1 and *in vitro* translated receptors in the presence of 0.1% DMSO as negative control, 1 µM each of E2 or ICI 182,780, and 100 µM each of the indicated compound. Co-resolved complexes were detected by fluorography, with 10% input of each receptor protein for comparison. This original gel image accurately reflects the order in which the samples were loaded and resolved; the complete uncropped image is in the Supplementary Information (see Fig. S1).

Since dSTACs appear to mimic oestradiol, it was hypothesized that they may also bind to SIRT1-ER transcriptional complexes as reported^23^. In support of this, pull-down assays showed that these compounds bound to these complexes comparably with oestradiol but they differed in binding strength; in contrast ICI 182,780 inhibited SIRT1 binding. Of the dSTACs tested, formononetin most strongly induced SIRT1 binding to both ER subtypes than E2 while isoliquiritigenin bound the least. These data support molecular models showing that formononetin has a higher affinity for ERα than E2, and other findings that isoliquiritigenin binds weakly to the ER^31,33,34^ (Fig. 3; see Supplementary Fig. S1**)**. The fact that dSTACs binding profiles to both ERα and ERβ were similar suggests that these data are veritable. Interestingly, all the molecules more preferentially recruited SIRT1 to ERα than to ERβ, reflecting the lower levels of coactivation of this receptor subtype as previously reported^23^; however, this may be cell type-dependent. Paradoxically, dSTACs binding did not always correlate with their relative potencies in ER transcriptional activation, e.g. isoliquiritigenin induced the strongest ER activation but it bound most weakly to SIRT1-ER complexes. Conversely although quercetin bound more strongly than isoliquiritigenin, it was a comparatively weaker activator of gene expression than the latter. These results show that dSTACs differentially bind to SIRT1-ER complexes but binding *per se* may not directly correlate with their abilities to activate SIRT1-ER signalling.

### Resveratrol non-monotonically modulates ER coactivation by sirtuins

**Figure 4:**
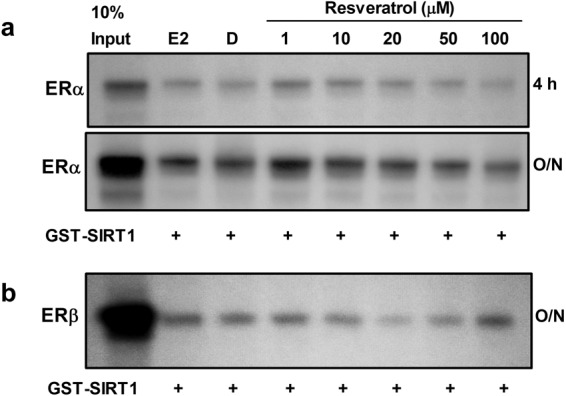
Resveratrol binds to SIRT1-ER transcriptional complexes. (**a**) Pull-down assay shows that resveratrol induces non-monotonic binding of SIRT1 to ERα. *In vitro* translated ERα was incubated with equivalent amounts of GST-SIRT1 (+) immobilized on glutathione sepharose beads and treated with DMSO (D) to detect ligand-independent binding, 1 µM E2 and with various concentrations of resveratrol. Bound complexes were co-resolved with 10% ERα input. The same gel was exposed to X-films for 4 h and then overnight (O/N). (**b)** Effect of resveratrol dosage on ERβ binding to SIRT1 as determined in (**a)**; note that only O/N exposure is shown as the signal from short (4 h) exposure was weak. Figure S2 in the Supplementary Information contains the full uncropped images.

**Figure 5:**
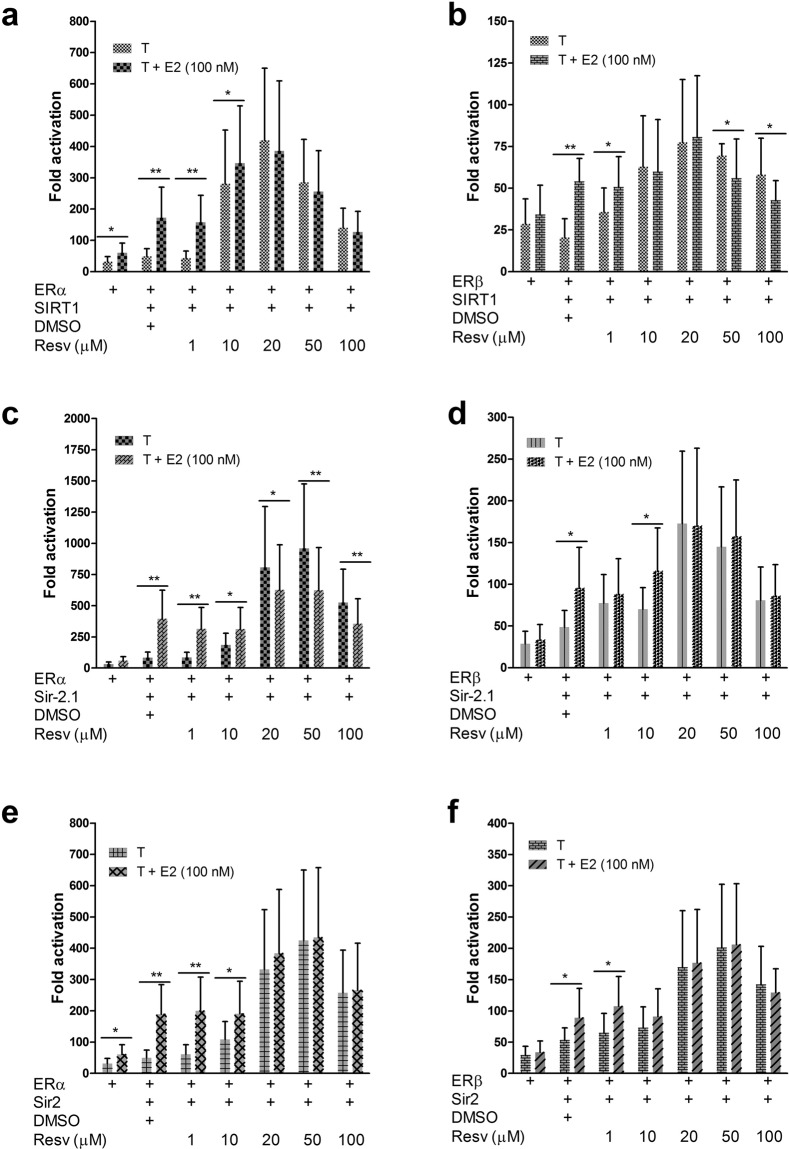
Resveratrol non-monotonically modulates sirtuin signalling through the ERs. (a, b) Oestrogenic and antioestrogenic effects of resveratrol on ER coactivation by SIRT1, **(c**,**d)** Sir-2.1 and **(e**,**f**) Sir2. Hep3B cells cotransfected with the sirtuins and ERα or ERβ and 3x ERRE.ERE-luc, were treated with DMSO (T) and with increasing doses of resveratrol (Resv) alone or in combination with 100 nM E2 to detect agonism or antagonism. Reporter activity was normalized as above and data points were plotted as means ± S.E.M (n = 3). Student’s *t*-test for differences between treatments with resveratrol alone or with E2 are significant at *(*P* ≤ 0.05) or very significant **(*P* ≤ 0.01); where not shown, *P* values are not significant.

**Figure 6:**
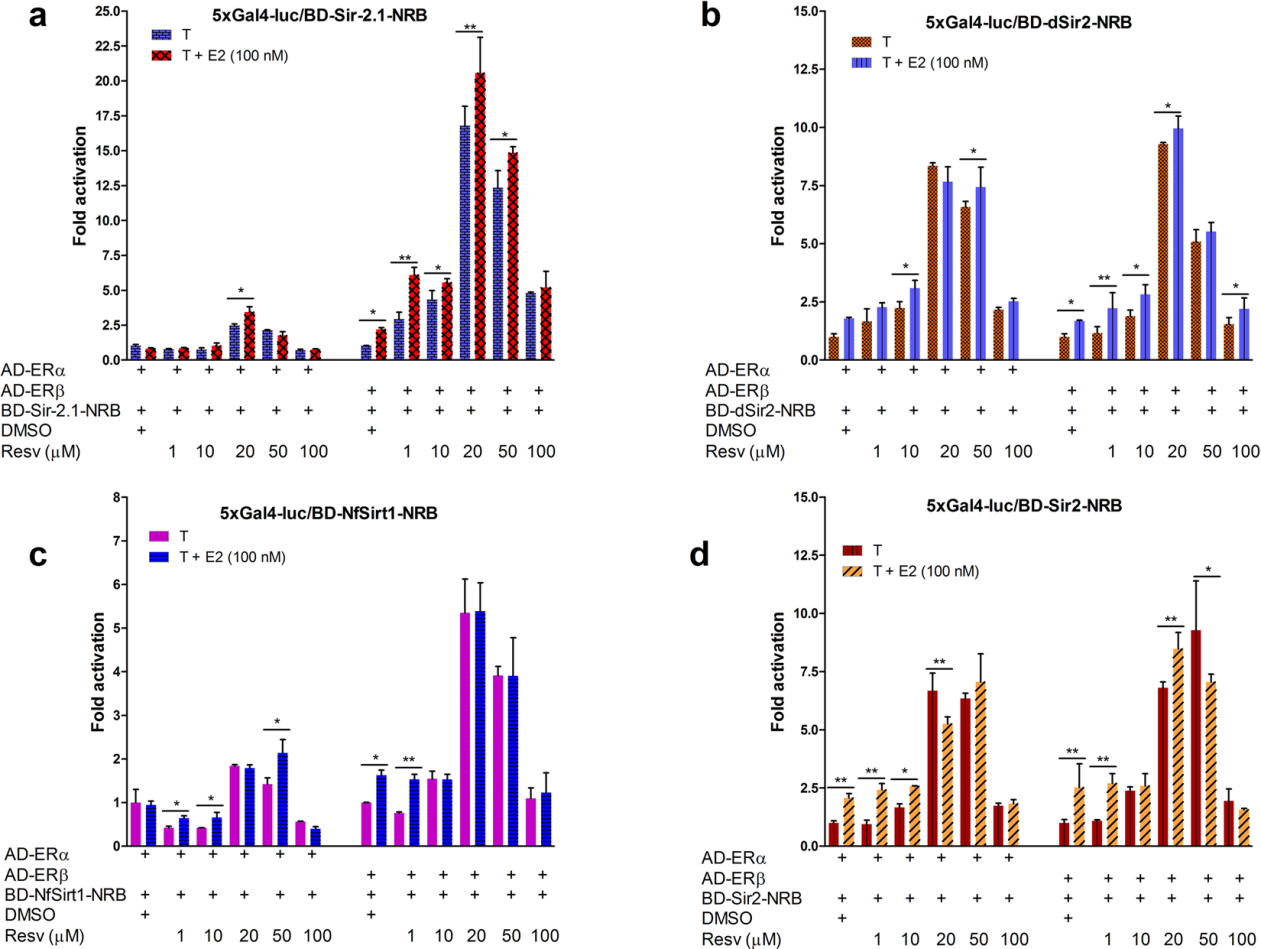
Resveratrol modulates interfacial interactions between the ERs and sirtuin NR-box (NRB) peptides of model organisms. Mammalian two-hybrid assays in HEK293 cells cotransfected with VP16 activation domain (AD) vectors of ERα or ERβ, the Gal4 reporter gene 5x Gal4-luc, and Gal4DBD (BD) vectors for Sir-2.1-NRB (**a**), Drosophila dSir2-NRB (**b**), *N*. *furzeri* Sirt1-NRB (**c**) and Sir2-NRB (**d**). Assays were performed as in Fig. 5 and fold activation was calculated from reporter activity in cells treated with DMSO. Note that this experiment used only 11-amino acid NR-box peptides^23^ from the cognate sirtuins whereas coactivation assays (Figs. 1, 2 and 5) used complete or near-full-length sirtuins. Error bars show means of 3 experiments ± S.E.M. Statistical differences between treatments (determined by paired Student’s *t*-test) are significant at *(*P* ≤ 0.05); where not shown, *P* values are not significant.

Among the dSTACs, resveratrol (3,5,4′-trihydroxystilbene; see Fig. 1b) has attracted the most attention due to its accessibility (e.g. in red wine) and demonstrable protection against metabolic, cardiovascular and neurodegenerative diseases^14–16^. In addition, it has been shown to increase lifespan in diverse organisms^8,35–37^. However like other dSTACs, the mechanism by which its signals are transduced by SIRT1 to confer protection or improve healthy ageing remains enigmatic. Resveratrol was therefore chosen for detailed examination to determine if and how it modulates ER coactivation by SIRT1. Firstly, binding assays showed that in low doses it behaved like oestradiol, promoting SIRT1 interaction with ERα while with increasing doses it reduced SIRT1 binding to the receptor (Fig. 4a; Supplementary Fig. S2). Interestingly it has been reported that unlike E2, resveratrol is a dynamic ligand that binds to the ER in two overlapping modes. Crystal structures indeed show that it binds ERα in two orientations whereas E2 binds in only one orientation^38,39^. It is unclear whether those findings explain why it bound more strongly to ERα than E2 at 1 µM. However consistent with previous data^23^, SIRT1 binding to ERβ was comparatively weak (Fig. 4b). These results were further reflected in activity assays which showed that in the presence of resveratrol, ERα coactivation by SIRT1 in Hep3B cells was much stronger than ERβ. Unexpectedly, resveratrol evoked a biphasic, non-monotonic dose-response typical of steroid hormone signalling^21,40,41^, resulting in inverted U-shaped transcriptional responses from both ER subtypes. Notably, ERα transcriptional activation inversely correlated with its binding because although SIRT1 bound most strongly at 1 µM resveratrol, only low levels of receptor activity was reported at that dosage, whereas maximal coactivation was seen at 10–20 µM but a reduction at ≥50 µM (Fig. 5a,b). These results strongly suggest that resveratrol may have a hormetic effect on sirtuin signalling. At 10 µM, it has been shown to extend yeast lifespan by ~70%^35^; hence it was notable that at the same dose, resveratrol was almost twice as potent as 100 nM E2 at enhancing ER coactivation by SIRT1 (compare bars 2 and 5 in Fig. 5a,b). In those low doses, resveratrol appeared similar to E2 in increasing ER coactivation but the converse was true at higher doses (50–100 µM); this is remarkably consistent with other findings^21^. Of note, the effects of E2 and resveratrol were not additive, indicating that they probably modulate gene expression by the same mechanism. Similar dose-response curves were also obtained with Sir-2.1 (Fig. 5c,d**)** and Sir2 (Fig. 5e,f) and although the latter was more tolerant than SIRT1 and Sir-2.1 at 50 µM, signalling through the ERs was repressed in all cases at 100 µM resveratrol. Although the latter result may have been due to cytostasis or cytotoxicity, it remarkably reflects the binding data which showed that SIRT1 bound weakly to ERα (but not ERβ) at that dosage. Previous data have also shown that the sirtuins share a conserved NR-box involved in ligand-dependent SIRT1 binding and coactivation of the ERs^23^. Mammalian two-hybrid assays between the receptors and the NR-box peptides of Sir2, Sir-2.1, dSir2 (*Drosophila melanogaster*) and fish (*Nothobranchius furzeri*) Sirt1, showed similar dose-response curves (Fig. 6a–d). These assays revealed that the NR-box peptides differed in ER selectivity and binding propensities possibly because adjacent residues also determine interaction specificity. Although these data were derived from mammalian cell cultures or with only the sirtuin NR-box peptides in the case of the two-hybrid assays, the subtle differences in resveratrol dosage optima may have important implications for assessing its effects on sirtuin biology in model organisms such as yeast and worms. Combined, these results strongly suggest that resveratrol may be oestrogenic in low doses but antioestrogenic at high concentrations.

### Dietary STACs modulate Sir-2.1 signalling through DAF-12

**Figure 7:**
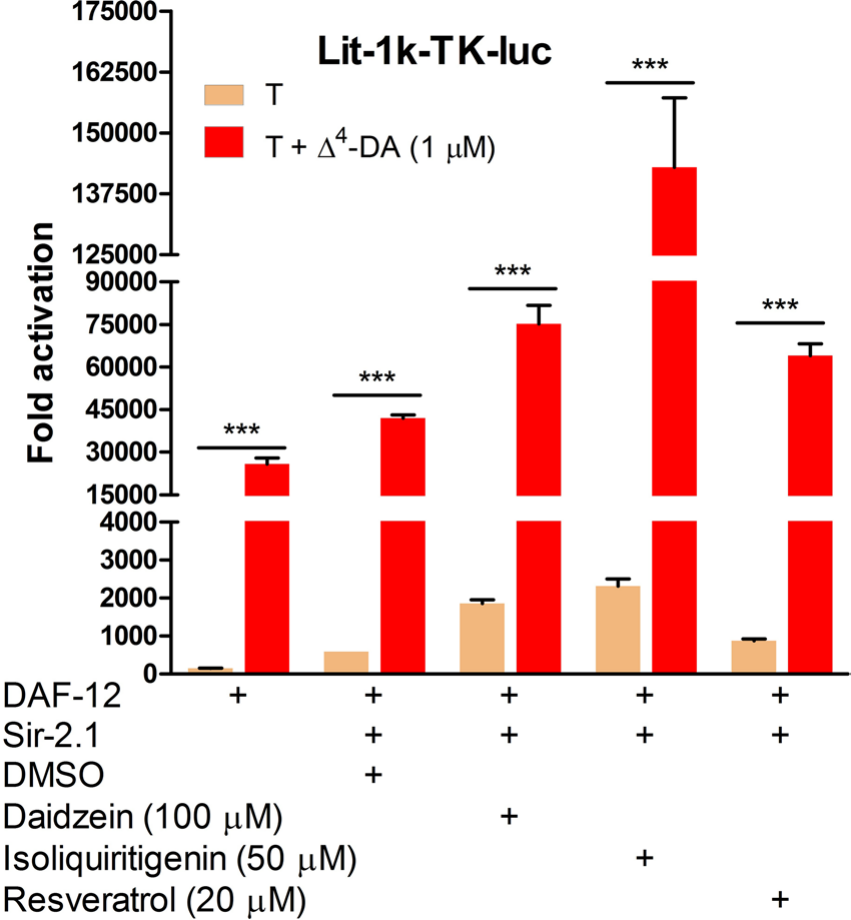
Sir-2.1 transduces dSTACs through the worm steroid receptor DAF-12. Hep3B cells cotransfected with DAF-12 and its reporter gene *lit-*1k-TK-luc and Sir-2.1, were treated (T) with DMSO control or with an exemplar dSTACs alone: isoflavone (daidzein), chalcone (isoliquiritigenin) and stilbene (resveratrol) or in combination with 1 µM Δ^4^-DA as indicated. Fold activation was calculated from the activity of *lit-*1k-TK-luc. Data sets were plotted as means ± S.E.M, (n = 3). Student’s *t*-test for differences in reporter gene activation by DMSO or STACs and STACs/Δ^4^-DA treatment are extremely significant (*P* ≤ 0.001).

Since resveratrol has been shown to delay ageing and to extend lifespan through yeast and metazoan sirtuins^35–37^, it was used along with related dSTACs (see Fig. 1b) to test if they could modulate Sir-2.1 signalling through the *C*. *elegans* steroid nuclear receptor DAF-12. This receptor has recently been shown to be coactivated by the sirtuins in the presence of dafachronic acids^23^ (DAs) - bile acid-like steroids that regulate worm developmental cycles, metabolism and lifespan^24–26^. Accordingly, DAF-12 and its reporter gene were transfected without and with Sir-2.1 into Hep3B cells and compared with DMSO, dSTACs differentially increased DAF-12 coactivation by Sir-2.1. Intriguingly, dSTACs synergized with Δ^4-^DA to further increase receptor transcriptional activation (Fig. 7). Notably in the absence of Δ^4^-DA, DAF-12 activation by dSTACs was much lower than with the ERs; e.g. isoliquiritigenin induced a ~4-fold increase in DAF-12 activity while it elicited ~ 200-fold increase in ERα activity (see Fig. 2a–d). While this selectivity could be due to differences in receptor cofactor requirements (because the assays were performed in mammalian cells), it is more likely because dSTACs do not mimic DAs as well as they do oestradiol (see Fig. 1a,b); this may partly account for their superior ability to ligand-independently activate the ER while DAF-12 activation was almost entirely ligand-dependent. Nonetheless these results suggest that both receptors may be functionally similar in their translation of dSTACs signals.

## Discussion

Sirtuin biology is fiendishly complex^1^; that of SIRT1 in particular involves several feedback loops and multiple reciprocal interactions with other proteins. Disentangling these interconnectivities has been difficult due to its role as a hub protein for diverse signalling pathways^5^, and an intrinsic capacity for autoregulation^23^. While its role in ageing/lifespan regulation remains controversial, its contribution to healthspan is not in doubt. Hence the use of dSTACs such as resveratrol and related allosteric activators for healthspan extension is of great interest. Although crystal structures have confirmed that these molecules bind directly to the STACs-AD^10,11^, it is unclear how their signals are transduced by SIRT1 or how those signals are translated for healthspan extension. The data reported here strongly suggest that dSTACs may impart health benefits including delaying ageing across taxa by exploiting their structural and functional mimicry of gonadal steroids to co-opt a conserved mechanism of sirtuin signalling through steroid receptors. This mechanism does not exclude allostery but indicates that dSTACs signals may come under endocrine control *in vivo*. As natural selective modulators of SIRT1 signalling through the ERs, dSTACs therefore have the potential to provide all the benefits of oestradiol such as protection from cognitive impairment and metabolic diseases^42–49^.

However abundant caution is required in ascribing biological relevance to these data because of potentially important confounding variables. Although it is recognized that SIRT1 and dSTACs prevent MARDs^2–4^, it is presently unclear from these data whether they do so through the ERs. Furthermore because dSTACs are poorly soluble in aqueous media, it is not known what fraction was absorbed and whether they underwent modification in cell culture. *In vivo*, their poor bioavailability and potential to undergo postprandial first-pass metabolism further complicates the biological validity of the data. For example, isoflavone methylation or glucosidation reduces their oestrogenicity^50^, whereas methylation of quercetin increases its potency^51^. In the case of resveratrol, although the native aglycone form can persist for hours postprandially, only small amounts are rapidly absorbed while a relatively large proportion gets glucuronidated within the small intestine^52^. It is unclear if those modified forms would be more or less bioactive than parental resveratrol in modulating SIRT1 signalling *in vivo*. Another possible layer of complexity is in the fact that some dSTACs can be metabolized by intestinal microbiota. For example, daidzein and resveratrol undergo biotransformation *in vivo* to equol and lunularin respectively but only in individuals with the requisite gut bacteria^53–55^. Since these metabolites appear to be more oestrogenic and also regulate food intake and body weight better than their precursors, it is tempting to speculate that these dSTACs may postprandially elicit stronger SIRT1-ER signalling than they show in these *in vitro* data.

Resveratrol was particularly remarkable on two levels. First, its dosage-sensitivity or non-monotonicity hints at a potential for hormetic signalling, an indication that it may evoke adaptive stress responses *in vivo* consistent with the xenohormesis hypothesis that dSTACs may be plant stress signalling molecules^56^. This may also be useful cautionary information because it suggests that contrary to popular lore, high doses of resveratrol may not be necessary to activate sirtuin signalling and may in fact be harmful, i.e. less is more. Instead, low dietary doses^57^ may suffice to elicit the hormetic responses required to prime defence mechanisms against incipient disease^58^. Secondly and more importantly, resveratrol elicited oestrogenicity in low doses but in large amounts it behaved like an antioestrogen, reducing sirtuin signalling through the ERs both in the absence and presence of E2. This janus-faced feature has been previously described as mixed agonism^22,59,60^, and may enable it to protect against a spectrum of diseases. For example, in low doses resveratrol induces gene expression signatures that mimic calorie restriction, delays ageing-related neurodegeneration, protects against obesity and non-alcoholic fatty liver disease, improves motor and cognitive functions and increases insulin-sensitivity in patients with type 2 diabetes^61–65^; all these effects remarkably overlap those of oestradiol^42–49^. In small amounts (5 µM) resveratrol also increased worm lifespan^66^. It is therefore interesting to speculate that in low doses it may mimic oestradiol, enabling it to up-regulate SIRT1 signalling through the ERs to confer all of the above health benefits as well as protect against other diseases associated with SIRT1 including the metabolic syndrome. Interestingly, SIRT1 has been shown to form transcriptional complexes with PGC-1α on regions of mitochondrial DNA that bind both ERα and ERβ^67,68^. This may partly explain why mice fed a resveratrol diet showed extended lifespan, increased mitochondrial biogenesis and metabolic capacity, and were protected from the damaging effects of high-calorie diets^61,69,70^. Of note, Sirt1 or Sir-2.1 ablation in cell culture and in animals severely attenuated responses to resveratrol and related dSTACs^36,71^ while the converse was true with sirtuin overexpression (see ref. ^12^ and references therein). Importantly, Sirt1 deletion reduced the ability of resveratrol to protect against skin cancer in mice^72^. Together these studies strongly suggest that these sirtuins may be critically important for transducing and translating the health benefits of dSTACs signals perhaps through steroid hormone receptors. In very low doses, resveratrol also rapidly and ER-dependently induces nitric oxide in endothelial cells^73^, indicating a possible mechanism by which it might protect against cardiovascular disease. Conversely, its antagonism of the ER in high doses hints at how resveratrol may reduce the risk of oestrogen-dependent cancers^74–76^; however, these effects may not be solely due to antioestrogenicity but also to its ability to induce cytostasis at those doses^60^. Hence these results are intriguing because they may partly explain the French paradox, i.e. the reduced incidence of cardiovascular diseases and certain types of cancer in some populations despite their consumption of high-fat diets^57,77^.

It may be important to note that the biphasic responses evoked by resveratrol and its non-monotonicity are typical of steroid hormones in general but of oestradiol in particular (see ref. ^32^ and references therein); this may prove consequential *in vivo*. At the molecular level, it could be speculated that in low doses, resveratrol may recruit ER coactivators such as SIRT1 and PGC-1α^23^. This may be supported by co-crystal structures showing that it induces a conformational change in ERα LBD that facilitates coactivator recruitment^39^. Alternatively, low-dose resveratrol may induce an open chromatin landscape permissive to SIRT1-ER coactivator complexes while in high doses it may either induce chromatin compaction that occludes these complexes or actively promote corepressor recruitment. It could also be that high-dose resveratrol induces a conformational change that inhibits ER binding/coactivation by SIRT1 (see Figs. 3 and 4). These assumptions may be consistent with reports that agonists and antagonists induce distinct ER conformations that respectively recruit coactivators and corepressors to the LBD^78,79^. Were similar conformers to be induced by low and high doses of resveratrol, they would explain its bifunctionality and the biphasic ER responses to it.

The ability of dSTACs to modulate ER subtype-selectivity and coactivation by SIRT1 may be teleologically important given that these receptors play vital physiological roles in health and disease^42–49^. Both ERs are expressed tissue-specifically suggesting that coactivation by SIRT1 may also be tissue-specific; for example, whereas ERα is strongly expressed in the liver compared with ERβ, the converse is true in the brain^19,49^. Oestrogen signalling through ERα regulates energy balance, food intake, bodyweight, glucose and lipid metabolism, and confers protection against osteoporosis, metabolic and cardiovascular diseases while ERβ is thought to protect against breast, colon and prostate cancers as well as neurodegeneration^42–49,80–85^. Notably the isoflavones increased ERβ coactivation by SIRT1 independently of oestradiol, suggesting how these dSTACs may protect against such conditions. Transcriptomic analyses have also shown that dSTACs induce distinct as well as overlapping gene networks regulated by ERα and ERβ^86^. Hence the ability of dSTACs to modulate SIRT1 signalling through both receptors independently of oestradiol could be important in non-reproductive tissues such as the liver, vascular endothelium, and adipose tissue but especially in skeletal muscle, brain and bone which markedly deteriorate with ageing. Dietary STACs may thus be most acutely beneficial in these tissues because their intrinsic oestrogenicity may subserve diverse oestradiol functions including neuroprotection^46,49,87–89^. These data may therefore provide clues to how dSTACs may reduce the incidence of MARDs.

What might underlie the ability of sirtuins to transduce diverse dSTACs signals through steroid receptors from evolutionarily distinct organisms? Phylogenetic evidence suggests that all extant steroid receptors evolved from a common ancestral ER (see ref. ^90^ and references therein) followed by ligand diversification from a primordial oestrogen or “paraestrol”^91^, i.e. steroid hormones including oestradiol and dafachronic acid evolved from a basic paraestrol template. Since this ancestral oestrogen predates phytoestrogens^90^, it is interesting to speculate that dSTACs mimic oestradiol because they may have been derived from the same polyphenolic backbone (see Fig. 1a,b), thus enabling them to co-opt sirtuin signalling through steroid receptors. Interestingly, ecdysteroid receptor activation by 20-hydroxyecdysone can also be modulated by dSTACs^92^, suggesting a common mechanism of signal transduction. Crucially, dSTACs are unable to activate the androgen, glucocorticoid, progesterone, and mineralocorticoid receptors. This selectivity appears to be due to the fact that ligands of these steroid receptors have a ketone group at their C-3 positions whereas ER agonists have a hydroxyl moiety. Both the number and positions of these hydroxyl groups determine ER-binding specificity^20,90,93,94^ (see Fig. 1a,b) which may partly explain why dSTACs preferentially activate the ER. In contrast and coincident with the loss of the ER in nematodes^90^, dSTACs are imperfect mimics of dafachronic acids which are structurally more similar to phytosterols than to phytoestrogens; this may partly explain why they could only residually activate DAF-12 in the absence of hormone. Hence dSTACs appear to be more specific to the ER than to other steroid receptors, indicating a primordial ligand-receptor pairing that persists in extant taxa. This selectivity may provide a basis to use them as templates to design functionalized and improved ER subtype-selective modulators^95,96^; e.g. ipriflavone is a synthetic derivative of daidzein used to prevent postmenopausal osteoporosis^97,98^. Resveratrol has also been modified or oligomerized into higher order molecular variants that bind differentially to ERα and regulate gene sets similar to those induced by oestradiol^99,100^. Interestingly the potencies of these oligomers directly correlate with the number of resveratrol repeats, leading to a spectrum of signalling outputs including tumour suppression, anti-inflammatory and lipid-lowering mechanisms^101–103^. Some synthetic forms have also been shown to be more bioactive than resveratrol in extending worm lifespan^104^ but whether that is mediated by Sir-2.1 is not known. It may therefore be possible to synthesize novel stilbenoids that more potently activate SIRT1-ER signalling than parental resveratrol.

In conclusion, the results shown here suggest that in addition to allosteric activation^8^, dSTACs such as resveratrol also ligand-independently enhance sirtuin coactivation of steroid receptors apparently by mimicking sex steroid hormones. Such exploitative mimicry and co-optation of sirtuin signalling could be especially important for hormone-independent ER coactivation in non-gonadal tissues most prone to ageing-related functional decline e.g. the skeletal muscle, brain and bone or in conditions where endogenous oestrogen synthesis may be inadequate e.g. the menopause. Hence this report may provide a new mechanistic insight into how dSTACs extend healthspan, and a basis to reimagine their potential as oestrogen substitutes to ameliorate or delay the onset of MARDs and to improve healthy ageing.

## Materials and Methods

### Chemicals

17β-oestradiol (E2/oestrogen), kaempferol, quercetin, naringenin, formononetin, fulvestrant (ICI 182,780) and analytical grade reagents were purchased from Sigma-Aldrich (Dorset, U.K.); isoliquiritigenin was purchased from Santa Cruz Biotechnology (Wembley, London, U.K.), and resveratrol was from Enzo Lifesciences (Exeter, U.K.). Δ^4^-dafachronic acid, daidzein and genistein were obtained from Cambridge Bioscience (Cambridge, U.K.) and SRT1720 was purchased from Selleckchem (Cambridge, U.K.). All compounds except formononetin were dissolved in DMSO and stored at −20 °C; formononetin was dissolved in ethanol and stored at 4 °C.

### Plasmids, transfections and reporter assays

All plasmids used in this study have been described elsewhere^23^. Cell culture media and antibiotics/antimycotics were obtained from Life Technologies (Paisley, U.K). Hep3B (ECACC 86062703) cells were obtained from ECACC (Porton Down, U.K.) and routinely cultured in DMEM (with 25 mM HEPES, GlutaMAX-1 and 4.5 g/l glucose), supplemented with 10% foetal bovine serum (FBS) and antibiotics/antimycotics. For transfections and treatments, cells were passaged onto 24-well plates (Corning, U.K.) and cultured in phenol red-free DMEM (Life Technologies) supplemented with 10% charcoal-stripped FBS (Sigma-Aldrich); this medium is hereafter referred to as PRF-DMEM.

For coactivation assays, Hep3B cells grown in PRF-DMEM were cotransfected with VP16-ERα or VP16-ERβ and the ER luciferase reporter gene 3xERRE/ERE-luc without or with 100 ng expression vectors of SIRT1, Sir2 and Sir-2.1. For DAF-12 coactivation, the cells were cotransfected with DAF-12 expression vector and its reporter gene *lit-*1k-TK-luc without or with Sir-2.1. Mammalian two-hybrid (M2H) assays were performed in HEK293 cells (ECACC 85120602) grown on 24-well BioCoat poly D-lysine plates (Corning, U.K.) and cultured in PRF-DMEM to determine the effect of resveratrol dosage on the interactions between VP16-ERα or VP16-ERβ and sirtuin NR-boxes; 5x Gal4-luc was used as reporter gene as previously described^23^. In both coactivation and M2H assays, 50 ng/well of pSVβgal (Promega, Southampton, U.K.) was cotransfected as internal control. For transfections, plasmids were diluted in phenol red-free Opti-MEM I (Life Technologies (Paisley, U.K) and transfected with X-tremegene HP reagent (Roche, U.K.) as instructed by the manufacturer in a total volume of 500 µl antibiotic-free PRF-DMEM. In both sets of assays, 500 µl fresh PRF-DMEM containing 200 nM E2 was added to the cells alone or together with 200 µM of select dSTACs ~12 h after transfection to final concentrations of 100 nM and 100 µM respectively; SRT1720 was used at a final concentration of 1 µM and ICI 182,780 at 100 nM. For cells cotransfected with DAF-12 and Sir-2.1, DMSO or dSTACs were added alone or in combination with 1 µM Δ^4^-DA. With resveratrol, various concentrations diluted in PRF-DMEM were added to the cells to give the desired final concentrations; DMSO was used as negative control at a final concentration of 0.1% in all assays. After a further 24 h-incubation, reporter assays were performed and luciferase expression was normalized to βgal expression levels as previously described^23^.

### SIRT1 expression, purification and interaction assays

Recombinant GST-SIRT1 was expressed from pGex6P-SIRT1 as previously described^23^. ERα and ERβ were expressed *in vitro* from pCITE4a-ERα and pCITE4a-ERβ vectors using the TNT T7 Quick Coupled Transcription/Translation system (Promega) and [^35^S]-methionine (Perkin Elmer, U.K.). For binding assays, GST-SIRT1 was immobilized on glutathione sepharose beads (Sigma-Aldrich) and washed 3x with 1x GST Bind/Wash buffer (Novagen, U.K.). The beads were resuspended in 20 mM HEPES, pH 7.5, 150 mM NaCl, 0.1 mM EDTA, 10% glycerol. Aliquots of glutathione slurry (~20 µl) containing bound protein were incubated in binding buffer [(20 mM HEPES, pH 7.5, 150 mM NaCl, 0.1 mM EDTA, 10% glycerol, 0.05% NP-40, and protease inhibitor cocktail (Roche)] in a total volume of 300 µl containing 5 µl ^35^S-labelled ERα or ERβ; DMSO (control), 1 µM each of E2 or ICI 182,780 and 100 µM of selected STACs were added. After nutating for 2 h at 4 °C, the beads were washed 3x with 1x GST Bind/Wash buffer and resuspended in 1 volume of 2x NuPAGE LDS sample buffer containing antioxidant. After heating for 10 min at 70 °C, immobilized protein complexes were eluted by a brief centrifugation at 13,200 rpm; 10 µl aliquots were resolved on 4–12% NuPAGE Bis-Tris gels (Thermo Fisher Scientific, U.K.); 0.5 µl (10%) of *in vitro* expressed receptors were run in parallel as input controls along with protein markers (Thermo Fisher Scientific). Gels were processed as described^23^.

### Statistical analysis

The statistical significance of differences in reporter gene activation by DMSO and different STACs was determined by Dunnett’s multiple comparison testing using GraphPad Prism version 5 for Windows (GraphPad Software, Inc., San Diego). Microsoft Excel (2010) was used where necessary, e.g. for spreadsheets and for Student’s *t-*test to compare steroid receptor responses to dSTACs alone and in the presence of hormone. Each experiment was routinely performed 2–4x and data points on graphs were plotted from replicate values as means ± S.E.M. *P* values ≤ 0.05 were considered to be statistically significant.

## Supplementary information

Supplementary information.

## Data Availability

Data reported in this article are available from the author upon reasonable request.
