# Supplementary material for: Sirtuins transduce STACs signals through steroid hormone receptors
Source: Sci Rep. 2020 Mar 24;10:5338. doi: 10.1038/s41598-020-62162-0 (PMC7093472; doi:10.1038/s41598-020-62162-0)
Supplement: Supplementary file 1 — Supplementary information. [file 41598_2020_62162_MOESM1_ESM.doc]

**Sirtuins transduce STACs signals through steroid hormone receptors**

Henry K. Bayele

Department of Structural and Molecular Biology, Division of Biosciences, University College London, Darwin Building, Gower Street, London WC1E 6BT, United Kingdom

Correspondence:

Email: [h.bayele@ucl.ac.uk](mailto:h.bayele@ucl.ac.uk)

Tel: +44 207 6792454

**Running title:** STACs modulate sirtuin signalling through steroid receptors

**
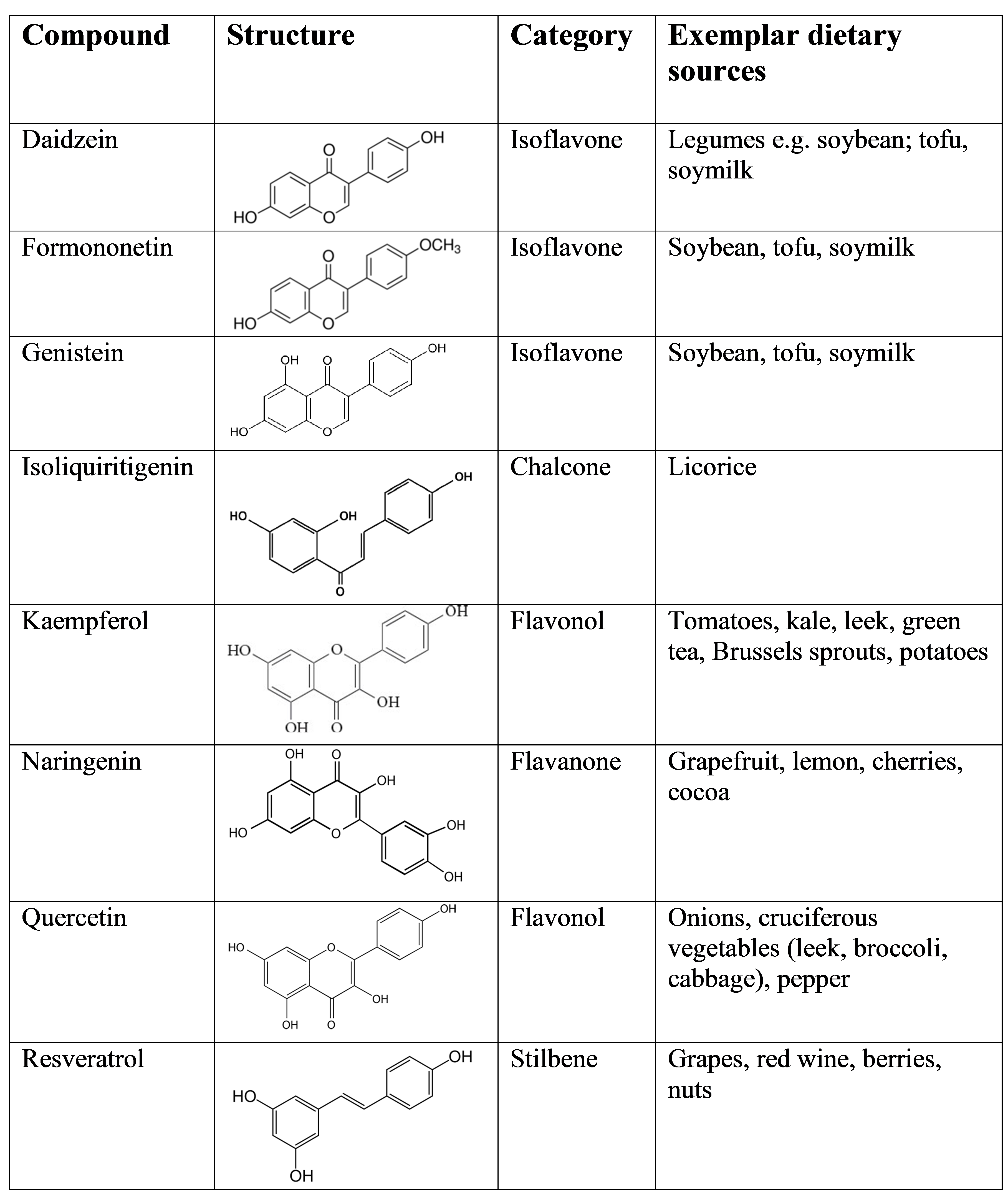
**

**Table S1.** Exemplar dietary STACs (polyphenols/phytoestrogens) used in this study: their structures, categories and principle food sources.

**
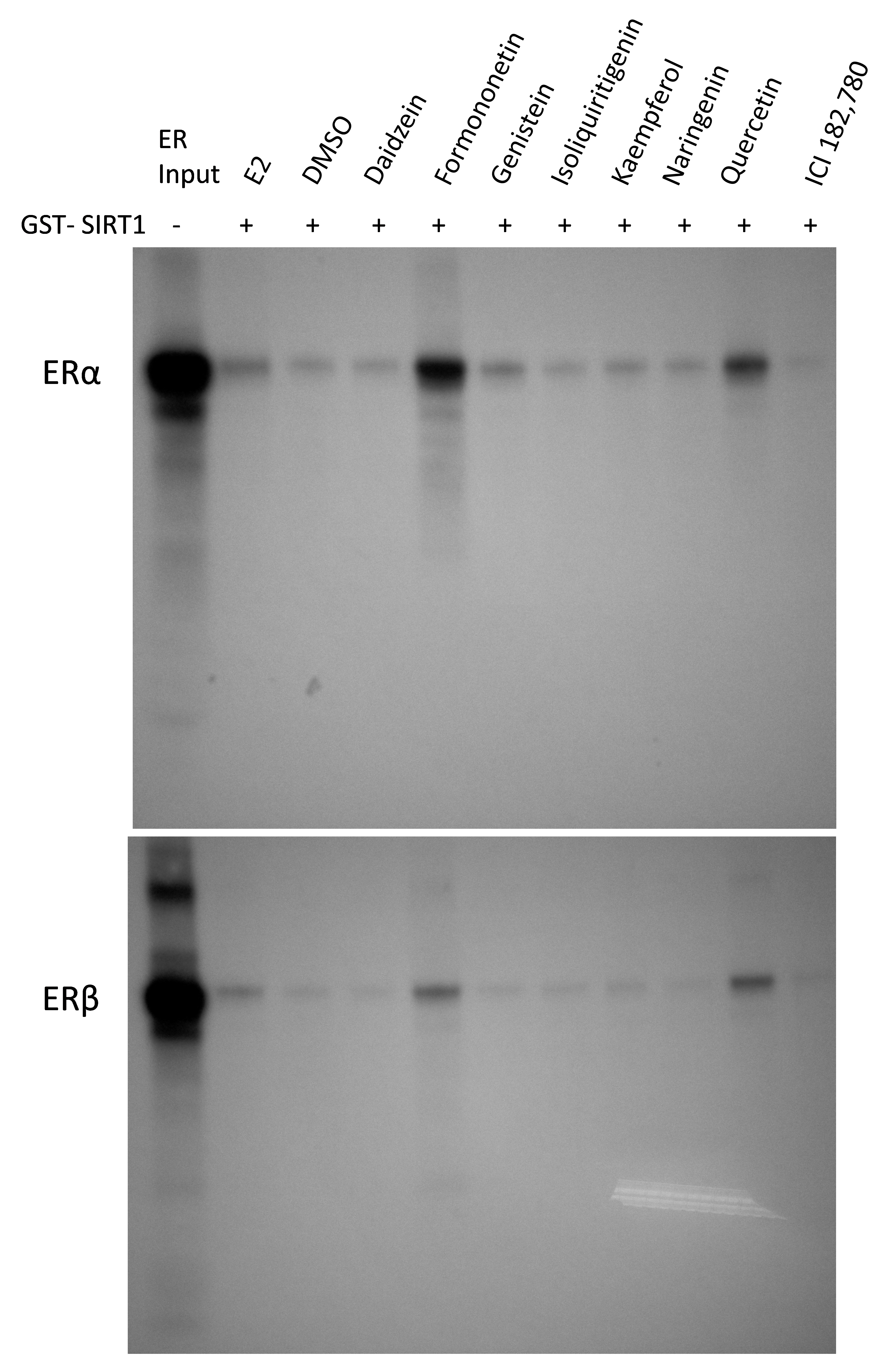
**

**Figure S1** Uncropped gel images of pull-down assay of STACs binding to SIRT1-ERα and SIRT1-ERβ transcriptional complexes (supplementary to Figure 3).

**
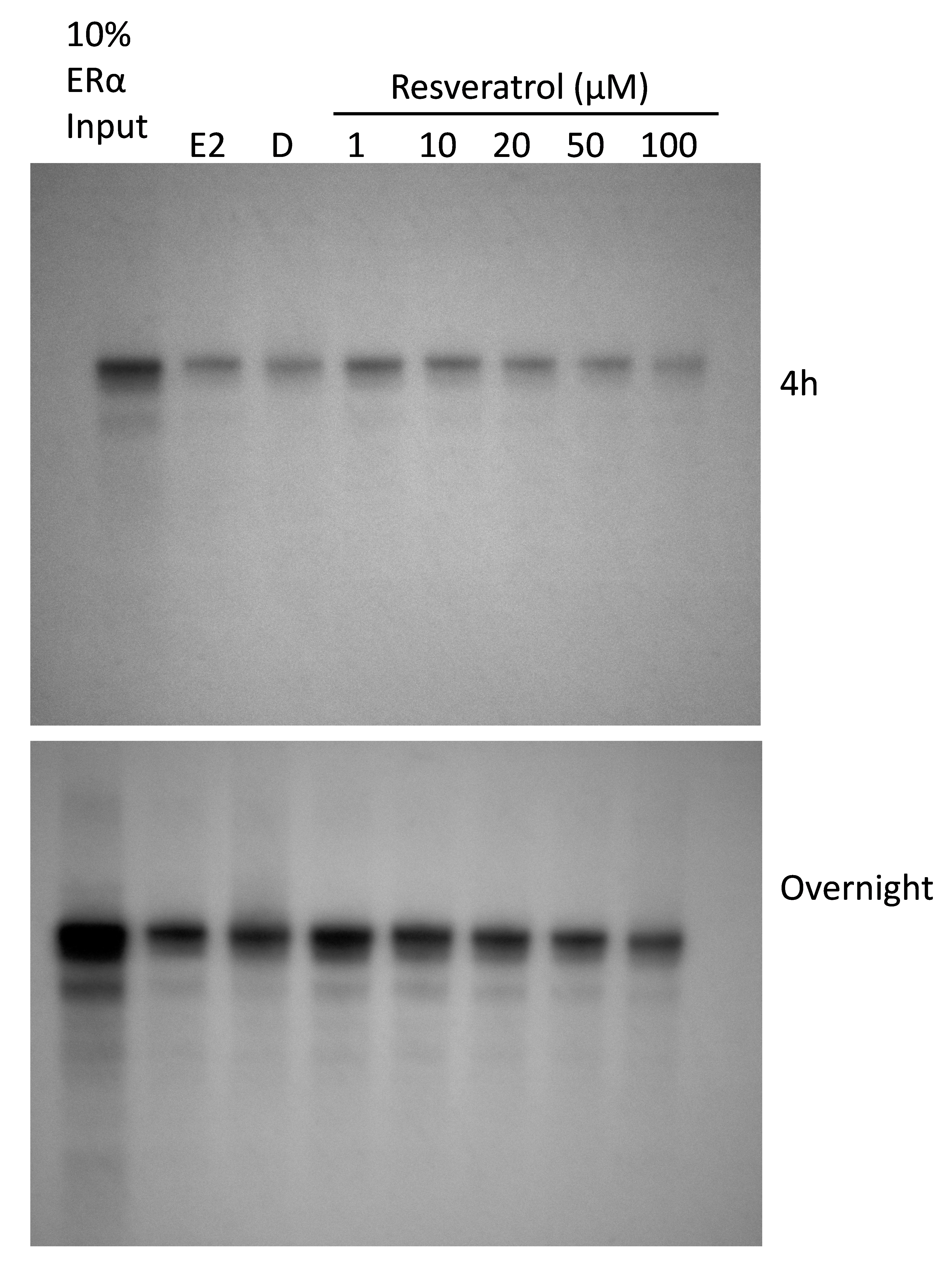
**

**Figure S2** Complete (uncropped) gel images of pull-down assay for resveratrol binding to SIRT1-ERα transcriptional complexes (see Figure 3). *In vitro* translated ERα was incubated with similar amounts of GST-SIRT1 and treated with 1 µM E2, DMSO (D) or various concentrations of resveratrol. Bound complexes were co-resolved with 10% ERα input and exposed to X-ray film sequentially for 4 h and then overnight.
